# The Principles of Medicine

**Published:** 1897-08

**Authors:** 


THE PRINCIPLES OF MEDICINE. An introduction to the study of special pathology. A text-book for students; being a course of lectures delivered to the classes of the Cleveland University of Medicine and Surgery, by E. R. Eggleston, M. D., Professor of Theory and Practice in the Cleveland University of Medicine and Surgery; late Professor in the Homœopathic Department of the University of Michigan. The Cleveland University of Medicine and Surgery, 1896. Price, $1.

This is an admirable little book for students of medicine. While it may be called a syllabus of Dr. Eggleston's lectures, it is much more. It is exactly what its name suggests, a summary of the principles of medicine, giving one a bird's-eye view of the various elements involved in disease-action.

The comprehensiveness of the treatment of the subject is shown by inspecting a few chapters. Thus in Chapter IX, on "The Elements of Disease," the whole organic system is divided into, 1. Contractile structures; 2. Nervous structures; 3. Secreting structures; 4. Blood and vascular system (circulatory structures); 5. Nutrition. All these are separately and concisely described, and the departures from the standard of action indicated.

Chapter X treats of nutrition. In this chapter the attention is drawn to hypertrophy and atrophy in their various modifications, degenerations. such as fatty hyaline, mucoid amyloid, colloid, calcareous, gangrenous, and caseous. Tubercle is treated separately. Temperature in health and disease is thoroughly yet briefly treated, and then there is an important chapter on the phenomena of disease. Of course there are chapters on diagnosis and prognosis, nature of disease, etiology, and so on. The whole of this material is included in a beautifully-printed book of 128 pages of square duodecimo size. so that it may be carried in the pocket and referred to as needed to clear up the mind upon these various points. It would not be amiss for the practitioner in active work to look at this book occasionally. and especially when he has a few minutes' leisure that cannot be usefully employed otherwise by reason of his situation away from his books and his desk. The writer acknowledges the benefit he has himself derived from it.

